# Toward Quality Standards for Brazilian Native Stingless Bee Honey Based on Physicochemical and Antioxidant Analyses

**DOI:** 10.3390/molecules31173128

**Published:** 2026-09-07

**Authors:** Maria Cristina Marcucci, Thais Bonagura, Thamires Bonagura, Geovani Moreira Da Cruz, Carolina Passarelli Gonçalves, Yuyang Guo, Ricardo Costa Rodrigues de Camargo, Alexandra Christine Helena Frankland Sawaya, Kai Wang

**Affiliations:** 1 Department of Biosciences and Oral Diagnosis, Institute of Science and Technology, São Paulo State University (ICT-UNESP), São José dos Campos 12245-000, SP, Brazil; geovani.moreira@unesp.br; 2 Municipality of São Bernardo do Campo–R. Samuel Sabatini, 50, São Bernardo do Campo 09750-901, SP, Brazil; thaisflorianonutricao@gmail.com; 3 Hospital Prof. Dr. Alípio Corrêa Netto–Ermelino Matarazzo, Alameda Rodrigo de Brum, 1989, São Paulo 03807-230, SP, Brazil; thamyyres_@hotmail.com; 4 Postgraduate Program in Chemical Biology, Federal University of São Paulo, Diadema 09913-030, SP, Brazil; carolina.passarelli@unifesp.br; 5 Laboratory of Geriatric Nutrition and Health of Ministry of Education, School of Food and Health, Beijing Technology and Business University, Beijing 100048, China; guoyuyang1026@163.com; 6 Researcher-Agroecology-Apiculture-Meliponiculture, EMBRAPA Environment, Jaguariúna 13918-110, SP, Brazil; ricardo.camargo@embrapa.br; 7 Faculty of Pharmaceutical Science, Campinas State University, R Candido Portinari 200, Campinas 13083-871, SP, Brazil; alexandra.sawaya@fcf.unicamp.br; 8 Institute of Apicultural Research, Chinese Academy of Agricultural Sciences, Beijing 100081, China

**Keywords:** native bee, honey, quality, antioxidant, regulation

## Abstract

Samples of stingless honey from six species of stingless bees collected at nine stations in different Brazilian states were analyzed by chemometric methods for moisture, impurities and solid residues, ash, pH, electrical conductivity, proteins, proline, identification of adulteration, presence of substances such as phytochemical analysis, color and levels of HMF. The color (C) varied between water white and dark amber. Proteins (mL) varied from 0.00 (**S5**) to 2.00 (**S4**). Lund test from 0.00% (**S5**) to 3.10% (**S6**), total phenols (TP%) from 0.04 to 13.45 mg/100 g GAE, total flavonoids from (TF%) 0.03 to 0.11 mg/100 g QE, and HMF from 0.00 to 14.28 mg/kg. There was an increased amount of PRO in **S7** and **S8**. **S1**, **S4**, **S7** and **S8** had the best antioxidant activity (EC_50_). A heat map showed that **S2** was the sample with the highest %Brix, %insoluble solids, %humidity and pH, but the lowest TP%. **S7** had the highest C, proline (PRO), TF%, EC_50_ and EC. **S4** had high %A and intermediate results between groups **9, 5, 6** and groups **1, 3, 8**. The sample honey **S6** showed a high content of TP (13.45 ± 0.98 mg/100 g of GAE). In contrast, stingless honey samples exhibited better DPPH scavenging activity (EC_50_: 21.45 ± 0.54 to 53.71 ± 0.24 mg/mL). The chemometric classification of stingless bee honey revealed that the physicochemical properties contributed significantly to the classification of stingless bee honey from different bee species. With all the information presented here, the results obtained and correlations from multivariate statistical analysis, we propose the creation of a standard for the technical regulation of Brazilian native bee honey, based on the literature and quality parameters described here.

## 1. Introduction

Native stingless bees existed before the colonization of Brazil [1,2,3,4]. Meliponinae are also known as indigenous bees. Knowledge from Kayapó and other indigenous tribes establishes the apicultural universe of native bees [3,5,6,7]. These bees have atrophied stingers, so they cannot sting when threatened; they belong to the subtribe Meliponinae, are classified in the Apidae family and are generally small to medium-sized [8,9,10,11]. Native stingless bees are responsible, depending on the ecosystem, for 40 to 90% of the pollination and are diversified into more than 300 species [12,13,14,15]. These bees are found largely in the tropical regions of the Earth, practically throughout Latin America and Africa, as well as Southeast Asia and Northern Australia. In Brazil, they are found from the state of Paraíba to Rio Grande do Sul [16,17,18]. There are also marauding bees, which live only by robbing the nests of other bees; these do not collect pollen from the flowers but transport the stolen pollen. The main one is the *irati* (*Nannotrigona testaceicornes*) or lemon bee (*Lestrimelitta limao*), characterized by exhaling a remarkable smell of lemon, although the honey of these bees is almost always considered toxic. They belong to the subfamily Meliponini [19,20,21]. The northeastern *uruçu* stingless bees (*Melipona scutellaris*), yellow *uruçu* (*Melipona mondury*), *mandaçaia* (*Melipona quadrifasciata*), *tiúba* (*Melipona compressipes fasciculata*), *jataí* (*Tetragonisca angustula*) and *manduri* (*Melipona marginata*) are social insects of great diversity and wide geographic distribution [22]. Their defense mechanism is indirect: it works through the construction of nests in hard-to-reach places, inside thick walls, tops of tall trees, deep cavities, and nests, which form a kind of crown, often facing downwards, which only allows the entry of one bee at a time, making it difficult for invaders to access the nest [23,24]. However, when these bees get tangled in hair and fur, they pinch with their sharp jaws and even penetrate the ears and nostrils, which is very uncomfortable [2]. Honey from stingless bees is more acidic than *Apis mellifera* honey and is produced in smaller quantities, about 1 to 10 kg of honey per year. On the other hand, *Apis mellifera* bees produce an average of 15 kg of honey per year [25,26,27]. Physicochemical parameters are methods that define and compare the composition and chemical substances of honeys for the purpose of defining a standard quality of the product. There is still no quality standard for honey from native bees established by the Ministry of Agriculture in Brazil, which raises doubts about the quality of the honey sold [28].

The quality of honey can vary depending on the species of bees; there are some with unhygienic habits that deposit their excrement where they store honey, for example, the northeastern *uruçu* (*Melipona scutellaris*). Native bees store honey inside their nests, inside their pots made of cerumen (the combs). There are environmental factors that can interfere with the quality of honey, such as agricultural pesticides and fungicides. Insecticides are used to prevent *Varroa destructor* mites; these environmental contaminants can be identified through laboratory analysis, and it is also possible to identify the botanical species from which nectars were collected [29,30]. Their low pH value (high acidity) potentially helps to increase the shelf life of honey because it decreases the proliferation of bacteria, but, on the other hand, the presence of undesirable fermentation may occur [26,31]. Honey from Meliponine is easier to ferment than honey from *Apis mellifera* [19,32,33]. Pasteurization is also used on honey and prevents fermentation. Honey from native bees is thinner and moister, so to prevent oxidation and fermentation, dehumidification is also used [32]. According to Brazilian law, the maximum moisture content in flower honeys or honeydews of *Apis mellifera* bees must be up to 20%, but honey from native Brazilian bees can have a moisture content of around 30%. There is still no legislation on quality standards; there is only legislation on the regulation of the activity of meliponiculture [34,35,36,37]. The National Council for the Environment (NCE) regulates that native wild bees can live naturally outside captivity, constituting part of the Brazilian wildlife [38]. The breeding and use of these bees must be authorized by the Brazilian Institute of Environment and Natural Resources (IBAMA) for commercial purposes, leisure activities, scientific research and for the consumption of honey or other products and the preservation of species [39]. To study native bees and their products in Brazil, it is necessary to obtain authorization from SISGEN [40,41]. In our research, we needed only the authorization from SISBio (Biodiversity Authorization and Information System), because we did not work with parts of organisms, DNA, proteins, or samples of native bees for technological development or bioprospecting purposes. In this case, an authorization of SisGen is necessary [41].

The production and commercialization of honey from native stingless bees is not yet regulated by Brazilian national law, only by regional rules for production. The purpose of the present work was to identify physicochemical patterns through the analysis of honey from several species of stingless bees and to propose parameters so that, in the near future, it will be possible to contribute to the drafting of a law for the regulation of quality control of honey from native Brazilian bees.

## 2. Results

### 2.1. Honey Samples and Bees

**Figure 1:**
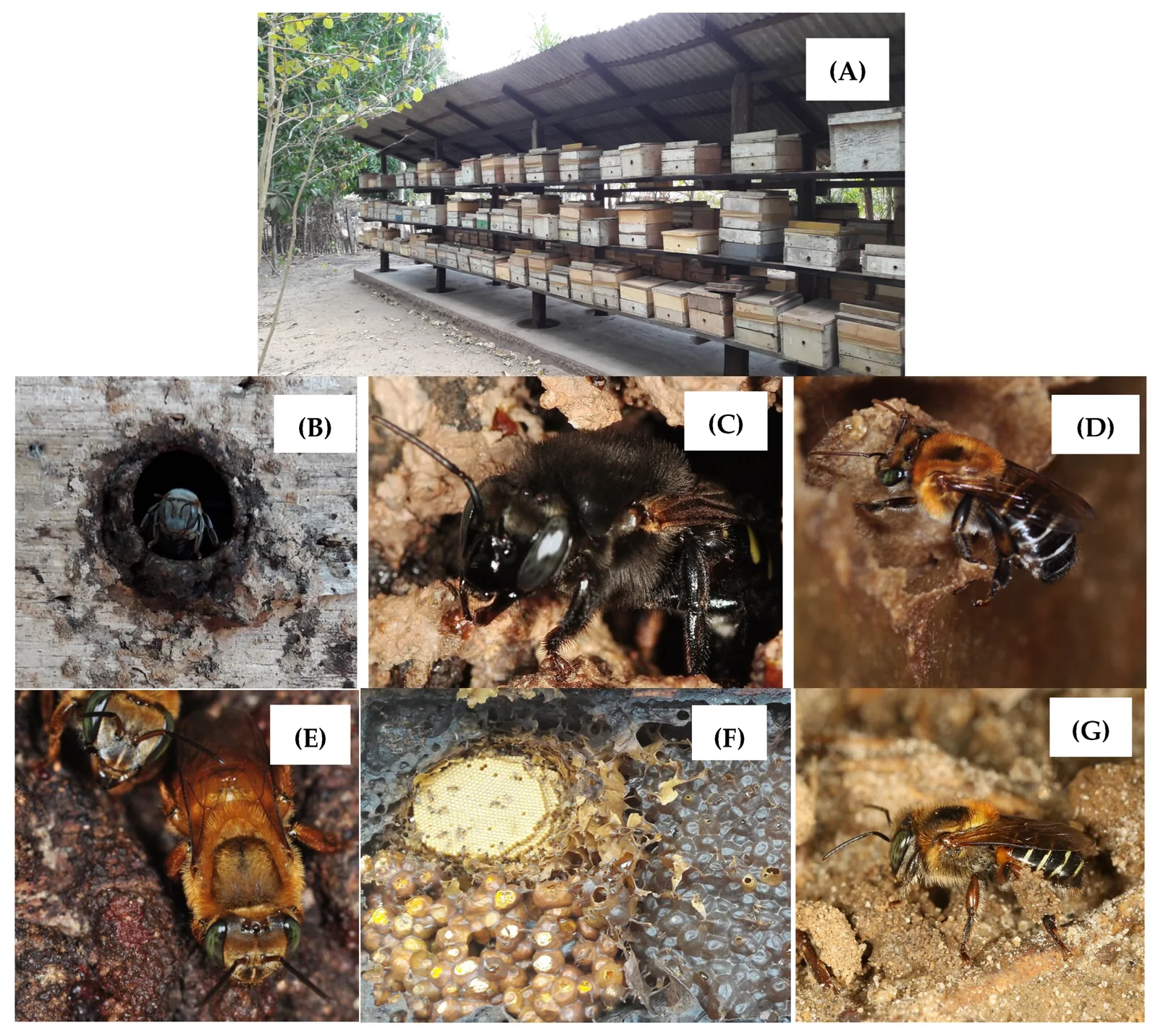
Photographs of native stingless bees that produced the honey samples studied herein. (**A**) Meliponary at Embrapa, (**B**) Tiúba (*Melipona compressipes fasciculata*), (**C**) Mandaçaia (*Melipona quadrifasciata*), (**D**) True uruçu (*Melipona scutellaris*), (**E**) Yellow uruçu (*Melipona mondury*), (**F**) nest of Jataí (*Tetragonisca angustula)* and (**G**) Manduri (*Melipona marginata)*. Photo: Ricardo C. Rodrigues de Camargo (meliponary from Embrapa, Jaguariúna, SP, Brazil).

The honey samples studied are from the bees shown in Figure 1. Geographic coordinates of the location: 570 m altitude, latitude 22° S, and longitude 47° W.

### 2.2. Determination of Physicochemical Parameters of Quality Control

The physicochemical properties of stingless bee honey from different regions of Brazil were shown in Table 1. The moisture (H%) of all the samples ranged from 29.78 ± 0.01 to 37.66 ± 0.03%. The *jataí*, *Tetragonisca angustula* **S7** honey from Pedreira, São Paulo State, had the lowest moisture (H%) of 29.78 ± 0.01%, and sample **S8** showed the same % of moisture (29.78 ± 0.05%). This sample is from Boqueirão de Leão, Rio Grande do Sul State (*jataí-Tetragonisca angustula*). The sugar content, indicated by the percentage of Brix, ranged from 62.00 ± 0.27% to 70.43 ± 0.28%.

The moisture content of honey is directly and inversely related to Brix. Brix degrees (Brix%) measure the total soluble solids content (mainly sugars) dissolved in honey. The higher the Brix, the lower the percentage of moisture (water) present in the honey [42].

The content of water-insoluble solids (%IS) indicates the amount of dirt present in the honey sample [43]. The results varied between 0.012% and 0.036% for the honeys analyzed (Table 1). The sample **S5** showed the highest content of IS (0.0360 ± 0.0007%).

Table 1 shows the percentage of ash (Aa) present in the honey samples, an indication of the mineral content [37,44,45]. The ash content ranged from 0.05% to 0.25%, except for **S4**, which showed a higher mineral content (0.37%) compared to the others. The pH is an important antimicrobial factor, which provides greater stability to the product and helps to control the development of microorganisms. All the honey samples studied (Table 1) are acidic, with values ranging from pH 3.00 to 3.36, which hinders the proliferation of microorganisms [46,47].

Regarding the results described in Table 1, only **S7** presented an electrical conductivity (EC) higher [48] than 1.370 mS/cm. The other samples have a lower value of electrical conductivity (0.133 to 0.796 mS/cm).

The color of honey (C) was determined in mm of Pfund. The colors varied from water white to dark amber in these samples (Table 1).

Normally in honey, a range from 0.6 to 3.0 mL of precipitate of albuminoid substances is found, evaluated by the Lund test (Figure S3). A deposit above 3 mL may be an indication of poor honey quality, and during the processing of the product there may be addition or loss of protein [49,50,51]. The precipitate of albuminoid substances (mL) in our honey samples was (**S1**) 1.00, (**S2**) 1.00, (**S3**) 1.66, (**S4**) 2.00, (**S5**) 0.00, (**S6**) 2.00, (**S7**) 0.50, (**S8**) 1.80 and (**S9**) 0.50. The precipitate of albuminoid substances, in milligrams and percentage (in parenthesis) in a total of 2 g of each honey sample, was (**S1**) 30.88 (1.54%), (**S2**) 31.07 (1.54%), (**S3**) 51.80 (2.57%), (**S4**) 61.62 (3.08%), (**S5**) 0.00 (0.00%), (**S6**) 61.84 (3.10%), (**S7**) 15.42 (0.77%), (**S8**) 55.70 (2.77%) and (**S9**) 15.94 (0.77%).

In the Lugol test, all samples presented negative results compared to a sample contaminated with 1% starch (Figure S4).

### 2.3. Determination of Total Phenols, Flavonoids, HMF and Proline

In Table 2, we can see that all samples have phenolic compounds (TP), varying between 0.04 and 13.45 mg/100 g of total phenols. Flavonoids (TF) are part of phenolic compounds, with antioxidant action, usually found in foods such as vegetables, flowers, grains, fruits, and wines [52,53].

All samples contained flavonoids (TF), ranging from 0.01 to 0.11 mg/100 g of honey (Table 2). Hydroxymethylfurfural (HMF) is a compound formed by the acid dehydration of monosaccharides (glucose and fructose), found naturally in honey. Table 2 shows the HMF content in the analyzed samples. Values between 0.00 and 14.28 mg/kg of honey were found, which indicates low HMF content. A typical chromatogram of HMF is shown in Figure S5. The presence of concentrations above 80 mg/kg of HMF in *Apis mellifera* honeys indicates overheating or adulteration of the honey with commercial glucose [35,54].

The free amino acid present in the largest quantity in honey is proline (PRO), produced by bees in the conversion of nectar into honey. As shown in Table 2, **S7** and **S8** showed the highest amount of proline with values of 2090.35 and 2208.23 mg/kg of honey, respectively [55].

### 2.4. Determination of Antioxidant Activity

The DPPH radicals, neutralized by antioxidant compounds, result in decreased absorbance at a wavelength of 517 nm [56]. We obtained the following results of EC_50_ (mg/mL): (**S1**) 54.95 ± 0.75, (**S2**) 149.65 ± 8.01, (**S3**) 799.04 ± 10.63, (**S4**) 53.71 ± 0.24, (**S5**) 211.48 ± 5.03, (**S6**) 112.50 ± 3.01, (**S7**) 21.45 ± 0.54, (**S8**) 45.11 ± 2.02 and (**S9**) 71.10 ± 3.21. The content of phenols and flavonoids in honeys is closely related to their antioxidant activity. Samples **S1**, **S4**, **S7** and **S8** showed the lowest EC_50_ values ranging from 21.45 mg/mL to 54.95 mg/mL, indicating moderate antioxidant activity. The remaining samples showed higher EC_50_ values, demonstrating weak antioxidant activity.

### 2.5. Chemometric Analysis

**Figure 2:**
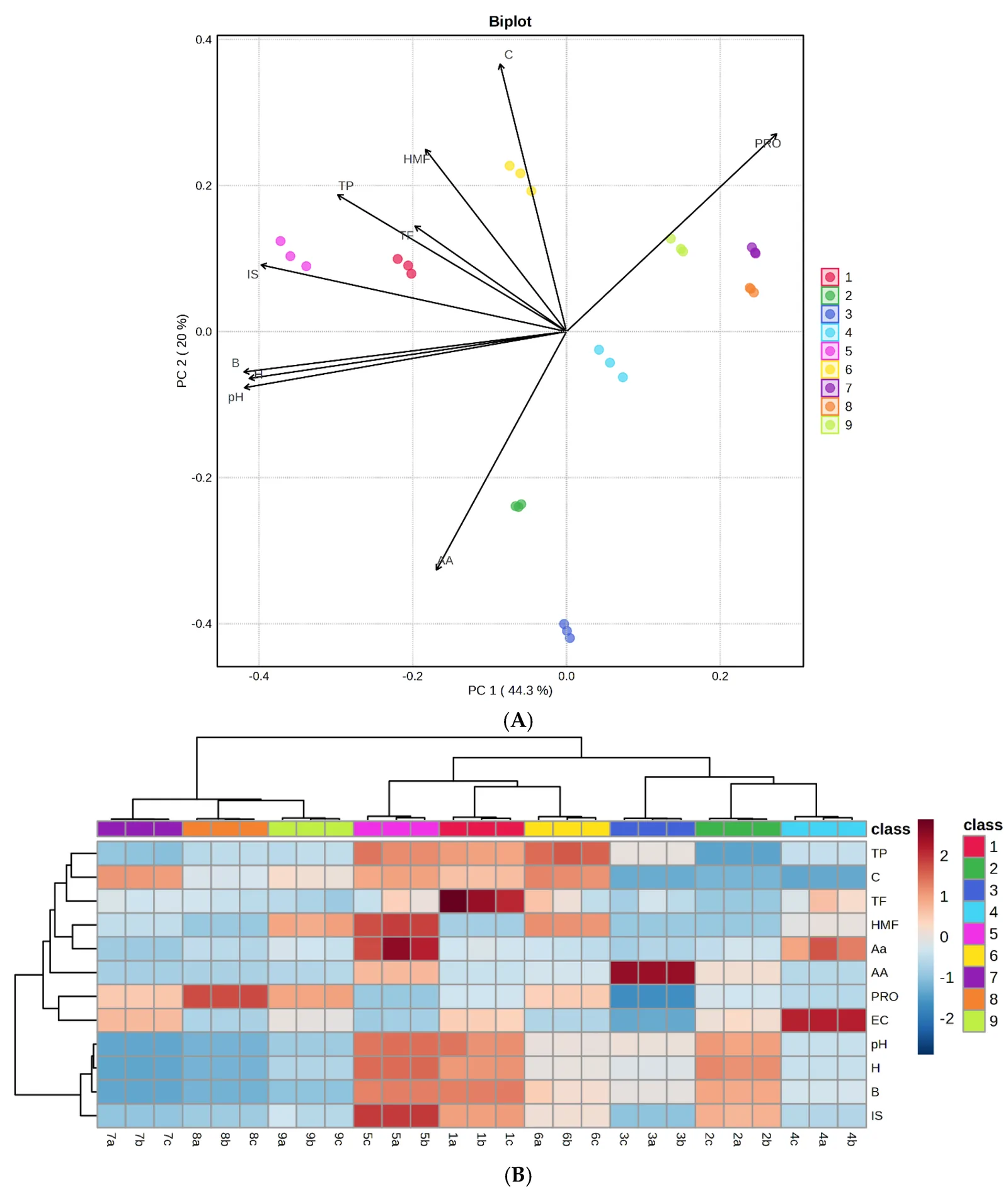
(**A**) Principal component analysis biplot showing the correlation between features and the variables (mean-centered and divided by the standard deviation of each variable). (**B**) Heatmap- using group averages, normalized data. (HMF—hydroxymethylfurfural; Aa—ash; TP—total phenolics; AA (EC_50_); EC—electrical conductivity; TF—total flavonoids; PRO—proline; B—°Brix; H— %Humidity; IS—insoluble solids).

A multivariate statistical analysis was performed with all data obtained. The results are shown in Figure 2.

The relation between variables and samples in the biplot (Figure 2A) is clearer in Figure 2B. In this case, a heatmap provides an intuitive visualization of a data table. Each colored cell on the map corresponds to a concentration value in our data table, with samples in rows and features/compounds in columns. Multivariate analysis showed the separation of the honey samples into three groups: group 1 (**S7**, **S8**, and **S9**), group 2 (**S1**, **S5**, and **S6**), and group 3 (**S2**, **S3**, and **S4**), which presented similar characteristics within the same group. The letters *a*, *b*, and *c* indicate the triplicate analyses. For example, honey **S3** showed a strong inverse correlation (dark red) with antioxidant activity, presenting the highest EC_50_ value and being the least active. The same occurred with sample **S5**, which also presented the highest amount of insoluble solids (IS). Honey **S2** was the one with the highest Brix, insoluble solids, humidity and pH, but the lowest total phenolic content, and stood out from the rest. Honey **S7** presented the highest color, proline, antioxidant activity and electrical conductivity. Group 1, with three samples (**S7**, **S8** and **S9**), presented similar results, with a very high content of proline (PRO). Group 2 (**S1**, **S5**, and **S6**) showed similar results for color (C), total phenols (TP) and flavonoids (TF). Group 3 (**S2**, **S3**, and **S4**) did not show strong correlations, other than color. However, honey **S4** presented high ash content and electrical conductivity.

Correlation analysis (CA) (Figure S6) was also carried out to reveal the correlation between the bee species from which the honey samples originated and their physicochemical properties, which were compared with the PCA results. The CA conducted to assess the similarity of stingless bee honey samples is shown in Table 3. As a result of the correlation analysis, as in the PCA analysis, there was a significant correlation between the physicochemical properties of honey from different bee species and stingless bees (Figure S6).

Regarding the multivariate statistical analysis, there was grouping among similar samples and linear correlations in the analysis data (Table 3).

## 3. Discussion

In a proposal for the creation of a technical regulation on identity and quality (TRIQ) for honey samples from native stingless bees in Brazil, a maximum value of 40% (*w*/*w*) moisture for fresh honeys was suggested [34]. A value of 56% moisture was found in Brazilian honey from *Melipona quadrifasciata* [57,58], and around 36% was reported by Bernhardt et al. [59]. Cruz et al. [32] reported 26% moisture for *Melipona scutellaris* honey. Muhammad and Sarbon [26] mentioned 29.2% moisture for *Trigona* spp. honey. The moisture content for *Tetragonisca angustula* honey was between 23 and 25%, as reported by Dos Santos et al. [60]. Grajales-Conesa et al. [61] reported around 20% to 26.8% moisture for *Melipona beecheii* honey in Mexico. Our results for moisture content (H) varied from 29.78 to 37.66%. The results presented here for the percentage of sugars (Brix) (62.00 to 70.43%) are also in agreement with those reported by Dos Santos et al. [60] and Chedao, Jehsu [62].

The content of insoluble solids (IS) described in the literature for *Tetragonisca angustula* honeys ranged from 0.04% to 0.09% [60]. We found slightly lower values, from 0.0120% to 0.0360% for the honey samples analyzed. Values of IS higher than those determined by legislation for *Apis mellifera* honeys [35] are generally related to failures during collection, the processing process during filtration and/or decantation of honey, as well as the habits of the bees that store the product [43,63].

The ash content (Aa) expresses the richness of honey in minerals and is a parameter widely used to verify its quality. The mineral content of honey is directly related to the type of soil and climatic conditions. The ash content in honey can be influenced by the incidence and diversity of floral sources during different seasons [64]. The low ash content in some samples may be characteristic of floral honeys, and the wide range of ash values may also indicate non-uniformity in management and/or harvesting techniques by beekeepers. Additionally, products with an amount of ash above the permitted amount (0.6%) may also indicate high levels of pollution [30,49]. Regarding ash content, a minimum of 0.03% was described for *Melipona scutellaris* honey and a maximum of 3.10% for *Tetrigona melanoleuca* honey [57]. The value of 0.51% was reported by Cruz et al. [32] for *Melipona scutellaris* honey and 0.83% for *Meliponula beccarii* L. [65]. Our findings (0.05 to 0.37%) agree with the references cited. This parameter (A) is also important for determining honey adulteration and is considered a marker of geographic and floral origin [65].

According to Cruz et al. [32], the pH of *Melipona scutellaris* honey samples varied between 4.72 and 5.86. Muhammad and Sarbon [26] found a pH value of 3.81 for honey from *Trigona* spp. For *Tetragonisca angustula* honeys, a pH range between 3.09 and 4.11 was reported [66]. Gadge et al. [45] proposed a pH range between 2.5 and 3.8 for native Malaysian stingless bees. Our results agree with parameters proposed by these authors and by Sharin et al. [64], who found a pH range between 2.71 and 3.17 for *Heterotrigona bakeri*, *Geniotrigona thoracica* and *Tetrigona binghami* honeys. The acidity of honey is an extremely important component, as in addition to providing chemical and sensorial characteristics, it contributes to its stability against the development of microorganisms. The pH value can be directly related to the floristic composition in the collection areas, since the pH of honey can be influenced by the pH of nectar, in addition to differences in soil composition or the association of plant species for the final honey composition [47,50,60]. Furthermore, a decreased pH of honey may be an indication of adulteration with relatively larger amounts of neutral substances, most likely sugar syrup, or adulteration of honey with water, which initiates fermentation, leading to the production of acids (acetic and succinic acids) responsible for reducing pH [67].

Our EC results varied between 0.133 and 0.796 mS/cm. Only **S7** presented a value of 1.370 mS/cm. The EC of honey is associated with the ash content (mineral content) and acidity, revealing the presence of ions, organic acids and proteins [58,68]. It was suggested that the assessment of electrical conductivity could replace the ash analysis, since the previous measurement is directly proportional to the ash content [58], which will be discussed later in this work. Abdi et al. [67] found a value around 0.390 mS/cm for honey from Ethiopia. The EC in honey varied from 0.126 to 0.917 for *Melipona beecheii* and 0.174–0.463 μS/cm for *Scaptotrigona mexicana* honeys, respectively [69]. In Brazilian species, EC ranges from 0.300 to 0.636 mS/cm for *Melipona subnida* Duke and 0.340 to 0.670 mS/cm for *Melipona scutellaris* Latrelle honeys [42]. The EC range reported by Dos Santos et al. [60] for *Tetragonisca angustula* honeys was from 0.174 to 0.189 mS/cm. All these values were below that required by the Codex Alimentarius Commission [70] for honey from *Apis mellifera* bees, which stipulates 0.80 mS/cm [71]. The importance of guaranteeing the authenticity and quality of honey is emphasized, even if the values observed are within the acceptable limit declared by the Codex Alimentarius Commission [70]. Pote et al. [72] reported an EC of *Tetragonula iridipennis* honey higher than that of *A. mellifera* honey (1.01–1.13 mS/cm. Similar values were reported by Ismail et al. [73], indicating that *Trigona* honey exhibited a higher EC (1.05 mS/cm) compared to *Apis* honey (0.62 mS/cm). High EC values, between 1.07 and 1.80 mS/cm and even higher (greater than 2.0 mS/cm), have been previously reported in honey from native stingless bees in Thailand [74] and Malaysia [75] according to Pote et al. [72] and Gebreyes et al. [76].

The color of honey was determined in mm of Pfund. The colors of these honey samples varied from water white to dark amber. The color of honey is associated with its botanical origin and minerals. The aroma and flavor are directly related to the color of the honey and predominantly depend on its floral origin [77]. De Souza et al. [42] found, for *Melipona subnida* Duke honey, colors such as 35.8 (extra light amber), 54.1 and 55.6 (light amber), and 95.4; and for *Melipona scutellaris* Latrelle honey, 55.9, 57.2 and 82.8 (light amber), and 103.4 (amber). According to the physicochemical parameters of *jataí* bee honey (*Tetragonisca angustula*) collected in the southwest region of Goiás, Rio Verde, Goiás, Brazil, the color identified in all samples was amber [59]. According to the literature [78], darker honey from *Apis mellifera* contains more minerals, dextrin and polyphenols, in addition to higher acidity than lighter honeys. The presence of cadmium, iron and lead produces a darker color, while aluminum and magnesium promote a lighter color [78]. An additional impact on color is the occurrence of crystallization caused by enzymatic reactions during storage and heat. Crystallized honeys typically have a lighter color [79,80].

The Lund test was based on the reaction of samples with tannic acid and the determination of the existence of albuminoid substances, offering information about possible falsification by some sweet substance added to water or some diluent added to honey [49]. In pure honey, after 24 h of reaction, a precipitate forms, oscillating between 0.6 mL and 3.0 mL of precipitate (Figure S1) [81]. In the presence of artificial honey or in artificial honey, no precipitate forms or only traces of precipitate appear [49,81,82]. Honeys **S3**, **S4**, **S6** and **S8** have a greater amount of albuminoid substances. However, **S1**, **S2**, **S7** and **S9** showed smaller quantities, but within the values described in the literature [49,50], except for **S5**, where there was no presence of precipitates.

The Lugol test is used to determine starch and dextrins, which react with an iodine solution in the presence of iodide (Lugol solution). The adulteration of honey results in loss of beneficial properties, reduces shelf life and makes the food more caloric [83]. In the Lugol test, when the sample is pure honey of floral origin, the solution does not react with the Lugol, maintaining its original color but when it is artificial honey or honey admixed with commercial sugar, the solution takes on a red or violet color with greater intensity depending on the greater presence of commercial sugar [82]. Our results showed that all samples have negative results compared to a sample contaminated with Lugol solution (Figure S2).

Phenolic compounds are secondary metabolites found in plants and some animal products [45]. They are known for their numerous health benefits due to their antioxidant properties, antimicrobial activity, anti-inflammatory effects and also have several other biological activities, including healing, antinociceptive, antidepressant, antidyslipidemic, antiobesity and prebiotic [84,85]. Honey from native stingless bees presented a wide variety of phenolic compounds, similar to traditional honey, such as flavonoids, phenolic acids and tannins [86]. These compounds are mainly derived from nectar and pollen collected by bees from various plant sources [87,88]. The chemical composition of *Melipona subnitida* products has been investigated through chromatographic analysis, demonstrating the presence of a variety of phenolic compounds, such as flavonoids and phenylpropanoids, among other classes of secondary metabolites [85]. The total phenolic content, described by Cruz et al. [32] for honey from *Mellipona scutellaris*, varied from 103.35 to 368.50 mg/100 g for samples stored at 5 °C and from 217.60 to 368.50 for the samples stored at 30 °C.

According to Gadge [45], the TP content in stingless bee honeys ranges from 5.43 ± 0.30 to 17.60 ± 0.20 mg GAE/100 g. Muhammad & Sarbon [26] reported a range from 0.12 ± 0.07 to 1.39 ± 0.11 mg GAE/100 g of honey from native bees in Malaysia. Our results are in agreement with those studies [26,45,46], varying between 0.04 ± 0.01 and 13.45 ± 0.98 mg GAE/100 g. The TF found by us ranged from 0.03 ± 0.01 to 0.11 ± 0.02 mg QE/100 g of honey.

HMF is formed when sugar in honey (fructose) is decomposed during an extended storage period or heating [89]. The Codex Alimentarius Commission [71] recommends an acceptable maximum of 40 mg/kg HMF and, in tropical regions, not more than 80 mg/kg of honey. When hot sugar syrup is added to honey, the fructose decomposes and elevates HMF in honey. The presence of HMF in honey leads to the browning and aroma characteristic of the Maillard reaction. This process is accelerated by temperature increase [54]. Thus, a higher HMF value of honey indicates adulteration, longer storage time, or heat-processed honey [67,90]. The honey from *Melipona quadrifasciata* L., evaluated by Ávila et al. [57], had an HMF content of 0.60 ± 0.05 mg/kg, complying with the parameters adopted in national legislation for *Apis mellifera* honey [35]. Subsequently, the same research group reported a range between 0.11 and 49.60 mg/kg of HMF in honey samples from different species of native stingless bees [58]. HMF was not detected in honey samples from *Melipona subnitida* or *Melipona scutellaris* [37], and for the second bee, 7.46 mg/kg was detected. The minimum value detected for this honey was 7.18 mg/kg, and the maximum was 8.22 mg/kg [91]. For *Melipona scutellaris* honey, the HMF value of 6.43 ± 0.15 mg/kg has been reported [32]. The HMF varied between 0.39 and 0.86 mg/kg in honey of *Tetragonisca angustula* L. [60]. According to Gadge et al. [45], HMF in honey from native bees can range from 12.64 to 15.0 mg/kg. Our results agree with these authors, varying between 0.00 ± 0.04 and 14.28 ± 2.78 mg/kg.

Proline (PRO) is one of the most abundant amino acids in some plants, and it is also preferred by bees. Reports indicate that this amino acid stimulates receptor cells, allowing bees to perceive taste. Proline is a source of energy for bee flight, being rapidly metabolized during the oxidation process, releasing significant amounts of ATP, which is the energy of cells. This property may lead to improved performance by bees in their search for food [92]. Proline is the main free amino acid present in honey produced by stingless bees, making it useful for the characterization of the botanical source because it is related to the floral source and the amount of pollen present in the honey [93]. Higher amounts of proline indicate a greater degree of maturity of the honey is also suggestive of its botanical origin, since the source of amino acids in honey comes from flower pollen [55]. An amount of 1723 mg/kg was found in honey from *Trigona laeviceps* [94]. The PRO levels in honey ranged from 20.5 to 4.6 mg/kg in *Melipona subnitida* and *Melipona scutellaris* honey. The honey samples from *Ziziphus joazeiro* Mart. (juazeiro) produced by both bee species also exhibited higher proline contents when compared to the other honey samples analyzed; however, honey from the same floral source exhibited similar proline contents [42,95]. Da Costa and Toro [96] found a range from 6.52 to 22.71 mg/kg of PRO in honey from *Melipona* spp. and in honey from *Plebeia flavocintica*, of 144.71 mg/kg. Our results varied between 260.86 ± 4.98 and 2208.23 ± 48.31 mg/kg in all analyzed honey samples.

The antioxidant activity (EC_50_) of honey has a direct and significant relationship with the plant and geographical origin of honey [97]. *T. angustula* honey showed antioxidant activity, reducing the DPPH radical. Promising results were also obtained by Gomes et al. [98] in honey samples collected in the western region of the State of Pará, Brazil, for *Melipona compressipes manaosensis*, *Melipona seminigra* and *Apis mellifera*, ranging between 11.98 and 70.51% in DPPH reduction [60]. The antioxidant activity of honey from native bees was evaluated by Damayanti et al. [99]. The scavenging activity of honey from *Tetragonula laeviceps* from Chantaburi and Trat Provinces in Thailand that was determined by DPPH (EC_50_) [100]. A similar study showed that the EC_50_ values of all stingless bee honey samples ranged from 14.29 to 90.63 mg/mL [101]. Another study reported that honey from native bees from Malaysia exhibited the highest antioxidant activity with EC_50_ values of 89.04 ± 0.83 mg/mL (8.90 ± 0.89%) [102]. The EC_50_ ranged from 76.04 ± 0.21 to 97.24 ± 2.53 mg∕mL for *Geniotrigona thoracica*, 32.58 ± 3.03 to 105.53 ± 10.78 mg∕mL for *Heterotrigona itama* [101]. A range between 4.55 ± 0.076 (*Apis dorsata*) and 64.271 ± 0.229 (*Geniotrigona thoracica*) was reported from honey from Indonesian bees [103]. Stingless bee honey could reach up to 45–95% higher antioxidant and biological activities than the traditional *A. mellifera* honey [58,99,101]. Our results for EC_50_ (mg/mL) varied from 21.45 to 799.04.

Physicochemical analyses allow for the characterization and traceability of the product. Therefore, based on the analyses performed here and other studies found in the literature, we concluded that it is possible to propose a quality standard for stingless bees’ honey to enable their commercialization in the formal market. A proposal of parameters to evaluate the physicochemical and microbiological quality of this kind of honey is detailed in Table 4.

Stingless bees are an integral part of Brazilian fauna, contributing to the biodiversity and natural resources. Therefore, their management is monitored and inspected by IBAMA [110]. The first proposed regulation for the creation of native bees in Brazil was Resolution # 346 of the National Environment Council (CONAMA), dated 16 August 2004 [111], which was recently revised by CONAMA, resulting in Resolution # 496, dated 19 August 2020 [112,113]. The responsibility for authorizing the use and management of stingless bees, previously exclusive to IBAMA, has been exercised collaboratively by the Federal Union, States, the Federal District, and municipalities since 2011 (Complementary Law # 140, of 8 December 2011). Currently, permits are granted by state environmental agencies [114]. For example, Brazilian rules for native bee honey vary by state: the Amazon region follows State Resolution (SR) CEMAAM #22 (3 April 2017); Bahia, SR #13.905 (29 January 2018); Goias, SR (*Ad referendum*) #007∕2017 CESMARH; Paraná, State Law (SL) #19152 (2 October 2017); Santa Catarina, SL #19171 (14 November 2013); Rio Grande do Sul, SL # 14763 (23 November 2015); Espírito Santo, SL #11077 (11 November 2019); Paraíba, SL # 11677 (4 May 2020); Maranhão, SL #11101 (6 September 2019); and Rio Grande do Norte, SL #10479 (30 January 2019) [16,34,113,115].

According to Menezes et al. [114], nowadays, there is a process at the federal level to establish a benchmark for the practice of this activity in Brazil. This process aims to incorporate important aspects such as the zootechnical focus that the activity can have, with shared responsibilities for registration and supervision of the activity between environmental and agricultural agencies, simplification of the registration of beekeepers and meliponaries, the establishment of actions to promote the activity, and the creation of a statistical database with information sharing between the competent sectors. For these reasons, and the growth of this activity, especially the production of honey from stingless bees, it is very important to establish quality parameters, as suggested in this work.

## 4. Materials and Methods

### 4.1. Honey Samples

The honey samples from the native bees were provided by the Brazilian Agricultural Research Corporation (EMBRAPA) Jaguariúna/SP. The collection of honey samples from native stingless bees (Meliponini) was carried out under the coordination of Dr. Ricardo Costa Rodrigues de Camargo, authorized by the Chico Mendes Institute for Biodiversity Conservation (ICMBio) through SISBIO license no. 51184, dated 22 September 2015 (Project: “Collection, research, conservation and sustainable use of bee genetic resources in agroecosystems and their products”), with Sisgen authorization # A903A71 (2022).

The samples were collected in September 2020 and stored in suitable jars to prevent contamination. These honey samples were kept refrigerated due to their high moisture content. Room temperature causes fermentation of the product and consequently loss of samples (Figure S1).

The origin of the honey samples was (**S1**) São Bento, Maranhão State (*tiúba-Melipona compressipes fasciculata*), (**S2**) Ilhéus, Bahia State (*mandaçaia-Melipona quadrifasciata*), (**S3**) Jaguariúna, São Paulo State (*uruçu-Melipona scutellaris*), (**S4**) Santa Rita do Sapucaí, Minas Gerais State (*jataí-Tetragonisca angustula*), (**S5**) Pedreira, São Paulo State (*mandaçaia-M. quadrifasciata*), (**S6**) Jaguariúna, São Paulo State (*uruçu amarela-Melipona mondury*), (**S7**) Pedreira, São Paulo State (*jataí-Tetragonisca angustula*), (**S8**) Boqueirão de Leão, Rio Grande do Sul State (*jataí-Tetragonisca. angustula*) and (**S9**) Boqueirão de Leão, Rio Grande do Sul State (*manduri-Melipona marginata*). The product was extracted with sterile syringes from the nests and was free of foreign matter such as wax, insect particles, combs, pollen grains and others.

Nine samples of honey of native Brazilian bees were studied (Figure S2). The moisture, impurities and solid residues, ashes, pH, electrical conductivity, proteins, proline, identification of adulteration, presence of substances such as phenols, flavonoids, antioxidant activity, color, and HMF of triplicate extracts of each sample were determined. The analytical procedures followed recommended methodologies [47,116].

### 4.2. Determination of Physical-Chemical Parameters

The moisture (%), Brix (%), ash (%), pH, electrical conductivity (mS∕cm), proline (mg∕kg) and HMF (mg∕kg) were determined according to Bogdanov et al. [48], water-insoluble solids (%) by AOAC [116], honey color (mm Pfund) by Bianchi [117] and Archilia et al. [118].

Briefly, in the Lund reaction, 2 g of honey and 20 mL of distilled water were mixed. The mixture was homogenized, and 5 mL of 95% tannic acid solution was added. Water was added to complete the volume to 40 mL, mixed well, and left to stand for 24 h. Afterwards, the amount of albuminoid substances in each sample was quantified in mL. In the presence of pure honey, a precipitate appears whose volume will depend on the floral origin of the honey. We determined the precipitate of albuminoid substances in milligrams and as a percentage in a total of 2 g of each honey sample (Scheme S1). With respect to the Lugol reaction, approximately 5 g of honey sample was weighed, and 10 mL of water was added. The mixture was stirred until the sample was completely dissolved, and 1 mL of Lugol solution (2% iodine and 6% potassium iodide in water) was added. In the presence of Lugol, adulterated honey presents a characteristic-colored reaction due to the presence of starch, corn syrup and dextrin, which does not occur in pure honey. The analyses were performed in triplicate [60,81,82].

### 4.3. Total Phenols and Flavonoids

About 0.2 g of honey was weighed, transferred to a 10 mL flask with about 5 mL of ultrapure water, and 800 μL of Folin–Ciocalteu reagent (Merck, Darmstadt, Germany) was added. About 1.2 mL of buffer solution (20% sodium carbonate; Synth, Brazil) was added, remaining at 20 °C for 2 h. The absorbance at 760 nm was measured. The phenol content was calculated from the standard gallic acid (Synth, Brazil) curve and expressed as gallic acid equivalents (mg/100 g GAE) [119]. For flavonoid content, about 2.0 g of honey was weighed and diluted in 8 mL of methanol (Synth, Brazil). It was filtered and placed in a 10 mL flask with 200 μL of 5% aluminum chloride (Synth, Brazil). The solution was left at 15 °C for 30 min. The absorbance was measured at 425 nm (Spectrophotometer Cary-50, Varian, Palo Alto, CA, USA). The flavonoid content was calculated from the standard quercetin curve (Sigma-Aldrich, St. Louis, MO, USA) and expressed as quercetin equivalents (mg/100 g QE) [119].

### 4.4. Antioxidant Activity

The antioxidant activity (EC_50_ is the dose that eliminates 50% of free radicals in mg/mL) was determined. The antioxidant potential was evaluated by the DPPH method (2,2-diphenylpicrylhydrazyl radical (Sigma-Aldrich, St. Louis, USA) (0.1 mM) by a kinetic method in ethanol (triplicate). Different concentrations of honey were kept in the dark for 30 min. The absorbance was measured at 517 nm in triplicate, and the EC_50_ (concentration that eliminates 50% of radicals) was determined by a linear regression [120].

### 4.5. Multivariate Statistical Analysis

In the multivariate statistical analysis (chemometrics), the processed data are represented by an X matrix. In the present study, the rows represent the honey samples of native bees, and each column (p) represents the variables (e.g., physicochemical data and antioxidant activity, EC_50_ in mg/mL). The T-matrix, called “scores,” represents the position of a sample in this new space. The V-matrix, called “loadings,” represents a matrix that describes the weight of each variable on this new coordinate axis (known as principal coordinates, PCs). MetaboAnalyst6.0 (https://www.metaboanalyst.ca/) was used, where sample variables (the individual results of triplicate extracts of each sample) were normalized by median and data autoscaled (mean-centered and divided by the standard deviation of each variable).

## 5. Conclusions

This study characterized Brazilian native stingless bee honeys through physicochemical, phytochemical, antioxidant, and chemometric analyses, contributing to the definition of relevant parameters for assessing the quality of these products. The results revealed distinct profiles among the samples, with variations in moisture, soluble solids, pH, ash content, electrical conductivity, color, phenolic compounds, flavonoids, hydroxymethylfurfural, proline and antioxidant activity. These findings demonstrate that stingless bee honey has its own identity and should not be evaluated exclusively according to criteria established for *Apis mellifera* honey. The integrated analysis of the data indicated that bee species and sample origin directly influence honey composition and quality. The presence of phenolic compounds and flavonoids, low HMF levels, the absence of adulteration according to the Lugol test, and chemometric groupings reinforce the need for specific parameters for the classification, quality control, and regulation of these honeys in Brazil. Therefore, the consolidation of specific quality criteria may benefit producers, researchers, regulatory agencies, and consumers by promoting greater traceability, standardization, safety, and recognition of the value of Brazilian meliponiculture products.

## Figures and Tables

**Table 1:** Some physicochemical parameters of honey from native bees *.

Honey	Moisture (%) (H)	Brix (%) (B)	Insoluble Solids (%) (IS)	Ash (%) (Aa)	pH	E. Conduct. (mS∕cm) (EC)	Color (mm Pfund) (C)
**S1**	30.38 ± 0.01	68.46 ± 0.10	0.0250 ± 0.0002	0.07 ± 0.01	3.04 ± 0.02	0.254 ± 0.010	51.51 ± 8.53 (Light Amber)
**S2**	34.18 ± 0.05	66.00 ± 0.57	0.0300 ± 0.0001	0.06 ± 0.01	3.17 ± 0.01	0.247 ± 0.015	7.65 ± 1.00 (Water white)
**S3**	30.33 ± 0.02	69.13 ± 0.41	0.0120 ± 0.0010	0.05 ± 0.01	3.36 ± 0.01	0.149 ± 0.003	6.34 ± 2.47 (Water white)
**S4**	29.82 ± 0.01	69.79 ± 0.58	0.0240 ± 0.0009	0.37 ± 0.06	3.00 ± 0.02	0.796 ± 0.007	5.38 ± 0.59 (Water white)
**S5**	33.30 ± 0.06	66.13 ± 0.41	0.0360 ± 0.0007	0.25 ± 0.03	3.20 ± 0.03	0.133 ± 0.002	58.15 ± 0.69 (Light Amber)
**S6**	29.88 ± 0.01	70.38 ± 0.21	0.0310 ± 0.0002	0.08 ± 0.01	3.03 ± 0.01	0.234 ± 0.002	101.61 ± 3.42 (Amber)
**S7**	29.78 ± 0.01	70.43 ± 0.28	0.0270 ± 0.0001	0.09 ± 0.01	3.28 ± 0.02	1.370 ± 0.008	135.58 ± 2.91 (Dark Amber)
**S8**	29.78 ± 0.05	66.50 ± 0.62	0.0300 ± 0.0005	0.16 ± 0.02	3.15 ± 0.01	0.570 ± 0.006	116.21 ± 1.77 (Dark Amber)
**S9**	37.66 ± 0.03	62.00 ± 0.27	0.0350 ± 0.0003	0.17 ± 0.02	3.30 ± 0.02	0.594 ± 0.007	114.01 ± 6.53 (Amber)

* Letters in parentheses were used in chemometric analysis.

**Table 2:** Phenols, flavonoids, HMF, proline and antioxidant activity of honey samples *.

Honey	Total Phenols (TP)	Total Flavonoids (TF)	HMF (HMF)	Proline (PRO)
	mg∕100 g of GAE	mg∕100 g of QE	(mg∕kg)	(mg∕kg)
**S1**	6.79 ± 0.20	0.09 ± 0.01	0.29 ± 0.03	316.22 ± 36.75
**S2**	0.04 ± 0.01	0.01 ± 0.01	0.00 ± 0.04	349.86 ± 29.53
**S3**	6.65 ± 0.04	0.03 ± 0.01	0.00 ± 0.07	260.86 ± 4.98
**S4**	5.32 ± 0.05	0.07 ± 0.02	4.64 ± 0.25	556.25 ± 36.36
**S5**	7.11 ± 0.03	0.03 ± 0.01	7.30 ± 1.67	235.57 ± 6.18
**S6**	13.45 ± 0.98	0.05 ± 0.02	8.66 ± 2.62	640.58 ± 4.84
**S7**	5.81 ± 0.60	0.11 ± 0.02	5.83 ± 0.68	2090.35 ± 103.5
**S8**	9.92 ± 0.77	0.08 ± 0.01	0.00 ± 0.32	2208.23 ± 48.31
**S9**	7.61 ± 0.75	0.04 ± 0.01	14.28 ± 2.78	1348.75 ± 86.35

* Letters in parentheses were used in chemometric analysis. GAE is gallic acid equivalents. QE is quercetin equivalents.

**Table 3:** Correlations identified in chemometrics between the analyzed parameters.

Parameters *	Correlation (R) **	Linear Regression
Antioxidant activity, EC_50_ (1/AA) *versus* electrical conductivity (EC)	0.948	y = 29,058x + 57
Antioxidant activity EC_50_ (1/AA) *versus* color, mm Pfund (C)	0.932	y = 856.43x + 97.59
Antioxidant activity, EC_50_ (1/AA) *versus* total flavonoids (TF)	0.861	y = 2.41x + 0.02
Proline (PRO) *versus* antioxidant activity EC_50_ (1/AA)	0.771	y = 0.00001x + 0.0041
Electrical conductivity (EC) *versus* proline (PRO) (calculated using averages)	0.737	y = 1.44x + 196.37
Proline (PRO) *versus* total flavonoids (TF) (calculated using averages)	0.618	y = 0.00003x + 0.0335

* Calculated using the raw data. ** Pearson’s correlation coefficient (R) is calculated from the coefficient of determination R^2^. The square root of R^2^ gives the value of R.

**Table 4:** Proposal of physicochemical and microbiological parameters for the regulation of honey from native stingless bees in Brazil.

Physicochemical Characteristics ^#^	Parameters				Limit	Reference
Maturity	Reducing sugars (calculated as inverted sugar) (%)				Min. 45/Max. 76	
	Apparent sucrose (%)				Min. 0.4/Max. 8.5	[34]
	Humidity (%)				Min. 19/Max. 39	[104]
	a) Dehumidified honey				Max. 20 g/100 g	
	b) *In natura*, pasteurized or matured honey				Max. 40 g/100 g	
Purity	Diastase (Göthe)				Min. 0.09/Max. 33	[104]
	Insoluble solids in water (%)				Min. 0.03/Max. 4.5	
	Minerals (ash) (%)				Min. 0.04/Max. 5.0	
	Pollen				Presence of pollen	[105,106]
Deterioration	pH				Min. 2.9/Max. 5.6	[104]
	Free acidity (mEq/kg)				Min. 2.5/Max. 220	[34]
	Water activity (a_w_)				0.52 a 0.80	[28]
	Hydroxymethylfurfural (mg/kg)				Min. 0.2/Max. 72	
Other parameters	Electrical conductivity (mS/cm) Lund (mL) Proline (mg/kg) Total phenolic content (mg∕100 g of GAE) Total flavonoid content (mg∕100 g of QE) Color (mm Pfund)				Min. 0.10/Max. 1.50 Min. 0.5/Max. 2.0 Min. 300/Max. 2200 Min. 5.0 Min. 0.025 Light amber to dark amber	[28]
Honey should show no signs of fermentation, except in mature honey.						
Inorganic Contaminants (ppm)						
Arsenic (As) ≤ 0.30	Lead (Pb) ≤ 0.30	Cadmium (Cd) ≤ 0.10		Mercury (Hg) ≤ 0.00	Tin (Sn) ≤ 0.00	[107]
Microbiological Characteristics						
**Microrganisms**	**Tolerance for Indicative Sample**	**Tolerance for Representative Sample**				**Method of Analysis**
		**n**	**c**	**m**	**M**	
Coliform at 45 °C (NMP */g or mL)	10^2^	5	2	10	10^2^	[108]
Molds and yeasts (CFU **/g or mL)	10^4^	5	2	10^3^	10^2^	[108]
*Salmonella* in 25 g	Absent	5	0	Absent	---	[109]

^#^ Values may be higher or lower depending on the honey composition due to climatic conditions, botanical flora, environmental factors, processing, and storage. Minimum: Min. and maximum: Max. * NMP: most probable number. ** CFU: colony-forming units. n: number of units to be randomly collected from the same batch and analyzed individually; c: maximum acceptable number of sample moistures with counts between the limits of m and M. m: the limit that, in a three-class plan, separates the acceptable batch from the product or batch with acceptable intermediate quality; M: limit that, in a two-class plan, separates the acceptable product from the unacceptable product (values above M are unacceptable). Absent, according to Brazil [104]; in this case, M does not exist (---).

## Data Availability

Data are available upon request. cristina.marcucci@unesp.br.

## Supplementary Materials

The following supporting information can be downloaded at: https://www.mdpi.com/article/10.3390/molecules31173128/s1, Figure S1: System to collect honey samples from native bees; Figure S2: Samples of honey from native bees analyzed in this study; Figure S3: Precipitate of albuminoid substances (red circle) evaluated by the Lund test; Scheme S1. Representation of the pathway for calculating the amount of albuminoid substances (mg) and in % in honey, through the Lund reaction. Calculation procedure developed by our research laboratory; Figure S4. Change of color after reaction with Lugol reagent. As examples, samples S1, S2 and S3. All samples had negative results compared to a sample contaminated with Lugol; Figure S5. HPLC-DAD of a honey sample (5) indicating the peak of HMF at 3.1 min; Figure S6. Correlations Heatmaps between variables, that origin data are reported in Table 3.
