# Supplementary material for: Toward Quality Standards for Brazilian Native Stingless Bee Honey Based on Physicochemical and Antioxidant Analyses
Source: Molecules. 2026 Sep 7;31(17):3128. doi: 10.3390/molecules31173128 (PMC13567636; doi:10.3390/molecules31173128)
Supplement: Supplementary file 1 [file molecules-31-03128-s001.zip › molecules-4454192-supplementary.pdf]

Toward Quality Standards for Brazilian Native Stingless Bee Honey Based on Physicochemical and Antioxidant Analyses

Maria Cristina Marcucci[1*], Thais Bonagura[2], Thamires Bonagura[3], Geovani Moreira Da Cruz[1], Carolina Passarelli Gonçalves[4], Yuyang Guo[5], Ricardo Costa Rodrigues de Camargo[6], Alexandra C.H.F. Sawaya[7], Kai Wang[8].

*Corresponding author: Maria Cristina Marcucci.

Samples of honey from native bees were provided by the Brazilian Agricultural Research Corporation (EMBRAPA) Jaguariúna/SP, Brazil, collected by agroecology-apiculture-meliponiculture researcher, Dr. Ricardo C. Rodrigues de Camargo. The collection of honey samples from native stingless bees (Meliponini) was carried out under the coordination of Dr. Ricardo Costa Rodrigues de Camargo, authorized by the Chico Mendes Institute for Biodiversity Conservation (ICMBio) through SISBio license no. 51184, from September, 22, 2015 (Project: "Collection, research, conservation and sustainable use of bee genetic resources in agroecosystems and their products"). SISbio (Biodiversity Authorization and Information System) is the official ICMBio system (linked to the Ministry of Environment and Climate Change – MMA, Brazil) that manages license applications for scientific research, educational activities, and collection of biological material in Federal Conservation Units and caves in Brazil. (https://www.gov.br/icmbio/pt-br/servicos/servicos-do-icmbio-no-gov.br/autorizacoes/pesquisa-nas-ucs-sisbio).

The samples were transported from Jaguariúna to the University laboratory in appropriate packaging. The samples were collected using a liquid microaspirator used by dentists (Figure S1). This method is used to ensure quick collection and better suction of the honey, but also by syringes and directly from the pot honey. Handlers must be properly uniformed and equipped with caps and gloves to avoid any contamination. The product must be extracted and free of foreign matter such as wax, insect particles, honeycombs, pollen grains and mites that can trigger allergic reactions in susceptible individuals when ingested. Other insects can secrete dirt such as feces on the honey, causing fungi, bacteria, viruses, protozoa and helminths, contaminating the product.

The honey samples were stored in suitable jars with lids to prevent contamination and kept refrigerated due to the high moisture content. Room temperature causes fermentation of the product and consequently loss of samples.

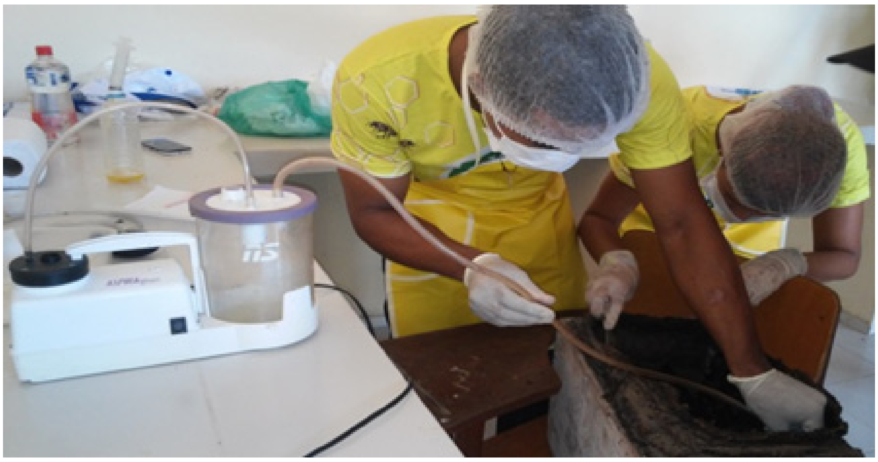

Figure S1. System to collect honey samples from native bees.
Photo provided by the researcher, Dr. Ricardo C. Rodrigues de Camargo, Jaguariúna/SP, Brazil.

Nine samples of honey (Figure S2) from native Brazilian bees were studied, one sample from each specie, and physicochemical parameters were determined, including antioxidant activity by DPPH.

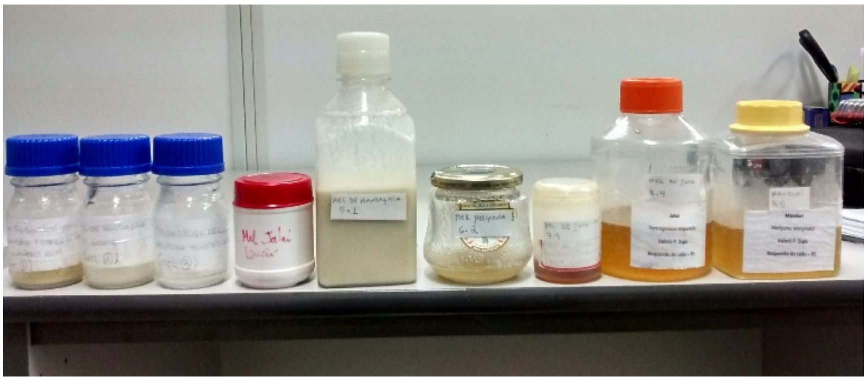

Figure S2. Samples of honey from native bees analyzed in this study.

The Lund reaction is based on the indication of the presence of albuminoid substances, components naturally present in honey. The precipitation of the proteins, in contact with tannic acid, occurs (Figure S3). This reaction indicates the integrity of the honey, whether it has been poorly processed or contains undesirable residues. When there is any adulteration present in the honey, a precipitate will not occur, or the quantity will be minimal [35] (Scheme S1).

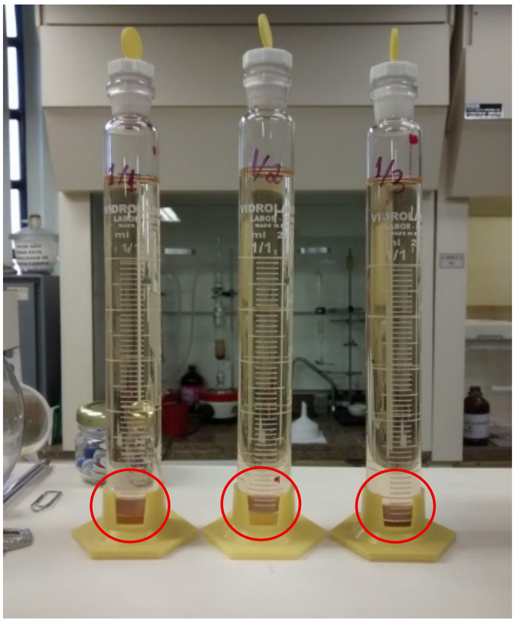

Figure S3. Precipitate of albuminoid substances (red circle) evaluated by the Lund test.

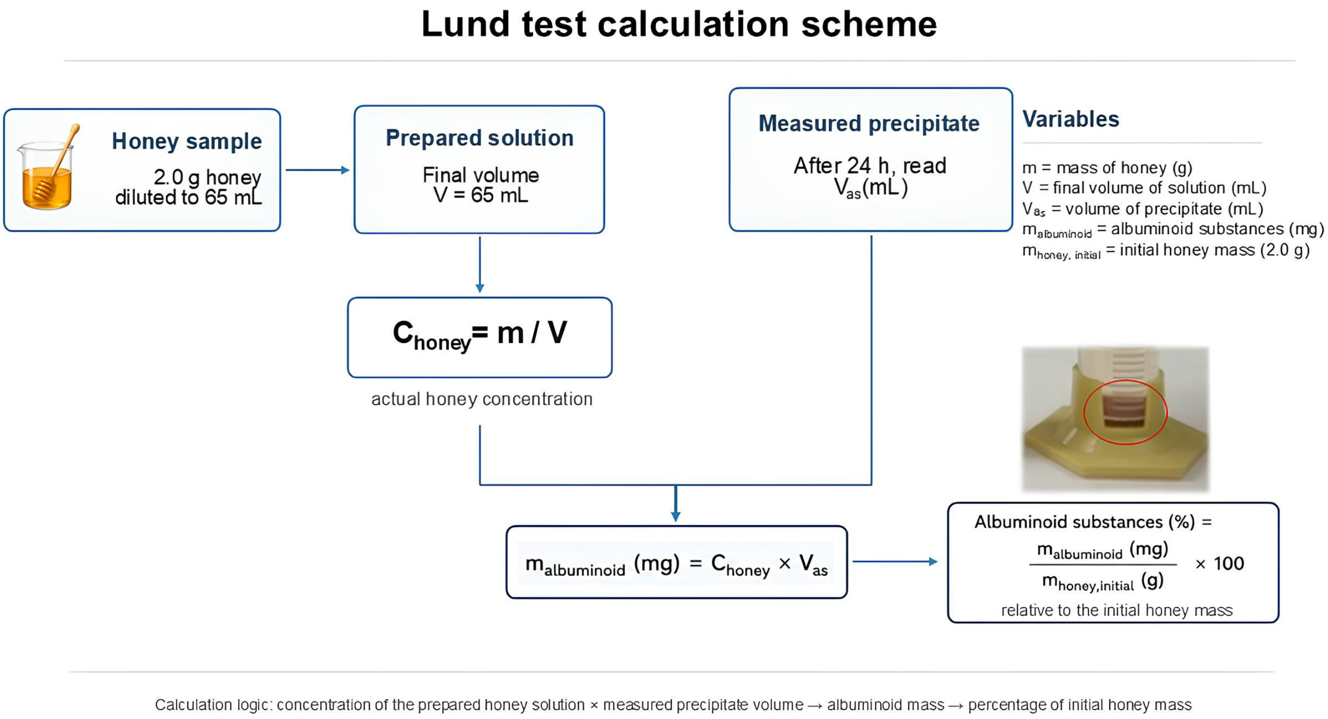

Scheme S1. Representation of the pathway for calculating the amount of albuminoid substances (mg) and in % in honey, through the Lund reaction. Calculation procedure developed by our research laboratory.

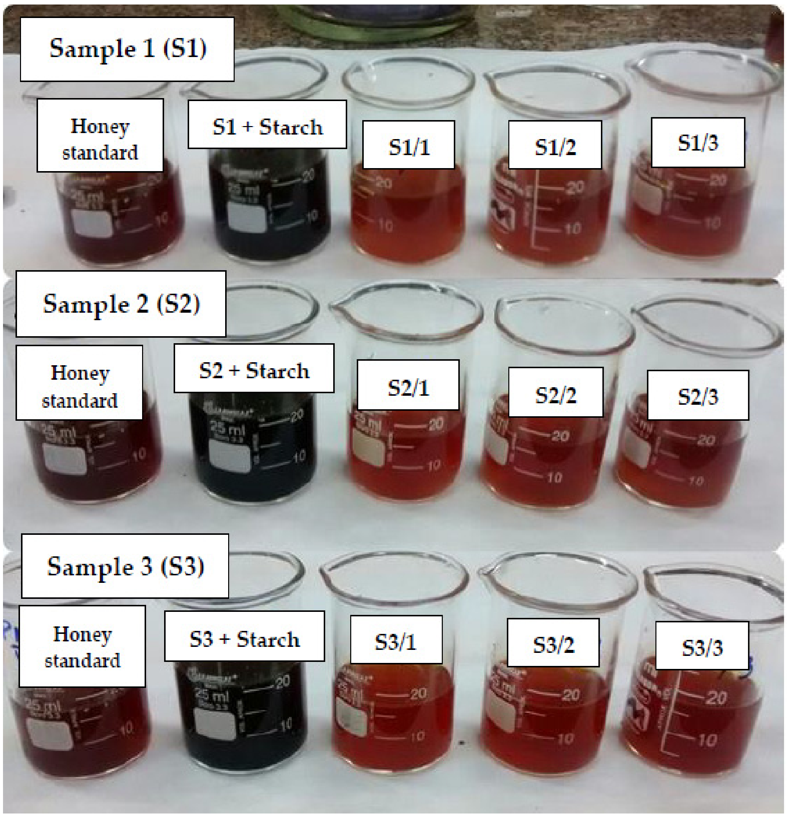

Figure S4. Change of color after reaction with Lugol reagent. As examples, samples **S1**, **S2** and **S3**. All samples had negative results compared to a sample contaminated with Lugol.

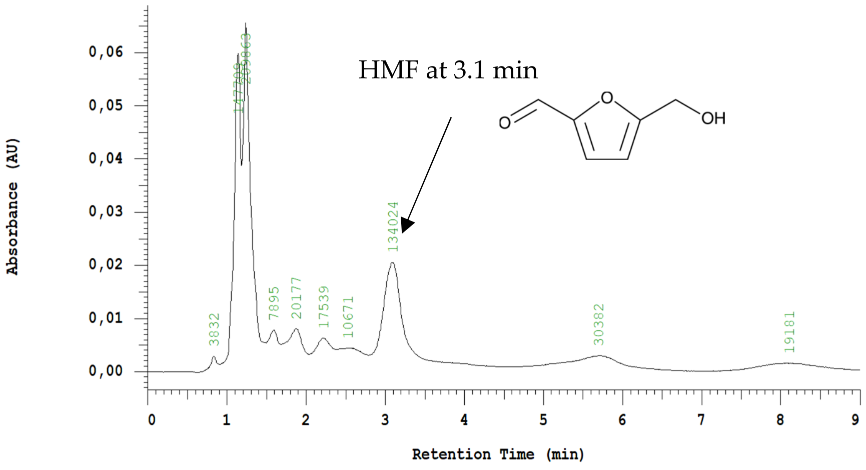

Figure S5. HPLC-DAD of a honey sample (**5**) indicating the peak of HMF at 3.1 minutes.

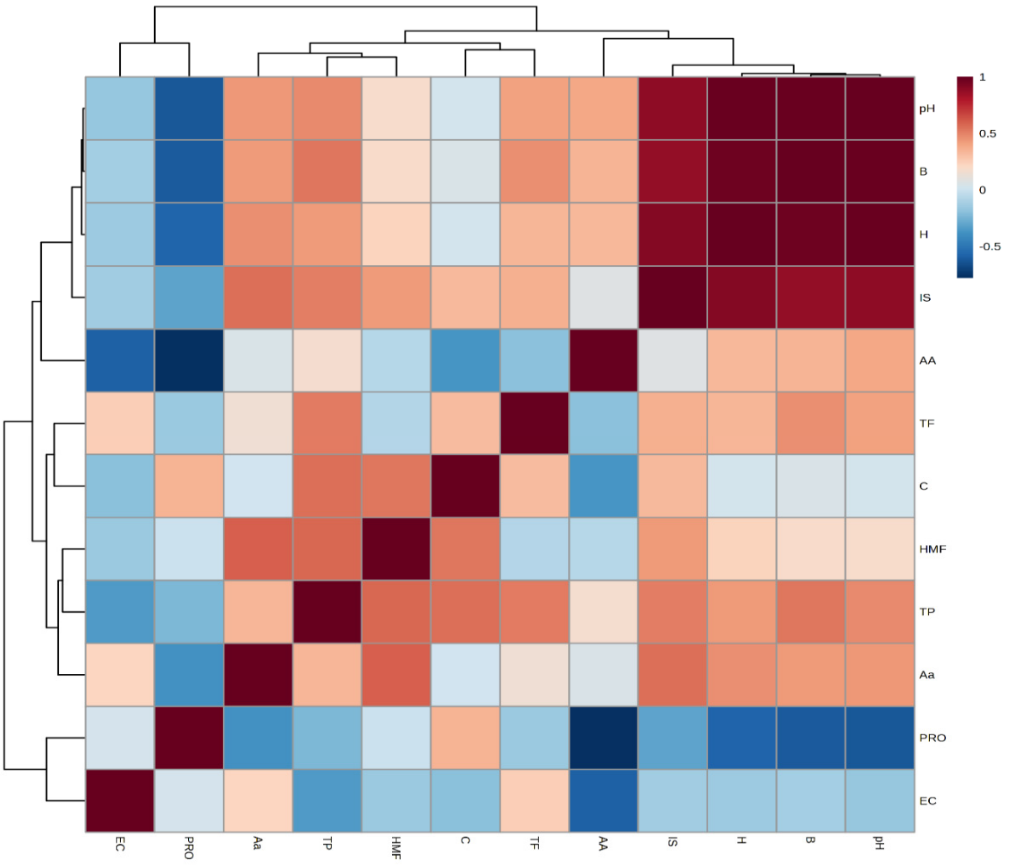

Figure S6. Correlations Heatmaps between variables, that origin data reported in Table 3.
